# Long-term cancer survivor awareness and learning preferences for cardio- oncologic risks: A qualitative study

**DOI:** 10.21203/rs.3.rs-8670497/v1

**Published:** 2026-01-27

**Authors:** Carolyn Smith-Morris, Marisel Ponton, Shelia Patterson, Arthur S Hong, Lauren Taylor, Kristine J Hahm, Dohyeong Kim, Vlad Zaha

**Affiliations:** The University of Texas Southwestern Medical Center; The University of Texas Southwestern Medical Center; Hoops Legends; The University of Texas Southwestern Medical Center; The University of Texas Southwestern Medical Center; University of Texas at Dallas; University of Texas at Dallas; The University of Texas Southwestern Medical Center

**Keywords:** Cancer Survivorship, Patient Education, Prevention

## Abstract

**Purpose:**

To understand general CVD risk awareness and learning preferences of cancer survivors, with particular attention to the learning needs of long-term (>5 year) survivors.

**Methods:**

We performed a qualitative sub-study of participants recruited from 14 community educational and health events in an underserved are of Dallas, TX from July 2023 – May 2024. Recorded interviews were transcribed verbatim, analyzed in two phases (framework analysis then systematic coding) to identify themes.

**Results:**

Twenty-six cancer survivors ranging in age from 30 to 82 (average age of 64.5 years) were interviewed, a large majority being long-term (>5 year) survivors and African American. Participants had diverse types of cancer, predominantly breast or prostate. Triangulation methods produced 3 themes: (1) Although a majority (73%) of participants self-reported knowledge about CVD risks after cancer treatment, narratives revealed a substantial lack of clarity about individual cardio-oncologic risk factors, such as the latent risks from chemotherapy or radiation. (2) Participants expressed trust and satisfaction with communications from their cancer treatment team with most (77%) stating that greater CVD risk understanding would not have changed their oncology treatment decisions. (3) For learning about CVD risks, participants preferred conversations with healthcare providers at multiple time points, before and after oncology treatment, as well as written materials that could be preserved and consulted later in the survivorship period.

**Conclusion:**

Survivors initially over-estimated their knowledge of CVD risks related to their cancer treatment and relied on their clinicians to raise concerns. Survivors’ awareness of CVD risks may be improved through targeted communications by clinicians, particular for long-term survivors, supplemented by durable reference materials. Greater attention to long-term CVD risk awareness in cancer survivors may improve CVD prevention among cancer survivors.

## Introduction

Cancer survivors are at increased risk for cardiovascular disease (CVD), due in part to the cardiotoxicity of cancer treatments and shared cardiovascular and oncological disease risk factors^1–6^. Cardiotoxicity has been linked to common treatments for breast cancer, including chemotherapy, molecularly targeted therapies, hormonal therapies, radiotherapy involving the heart field, and newer immunotherapies^7–10^. Also, men with prostate cancer are 4.5 times more likely to die due to other causes than cancer, mostly due to CVD.^11,12^ These differences in cardiotoxicity are compounded by social and economic factors that follow racial and ethnic patterns with higher prevalence cancers, such as breast and prostate cancer, having the widest disparities in cancer mortality along racial lines^13,14^.

Cancer survivors have limited awareness of their CVD risks and cardio-oncology care resources^15^ despite more than a decade of calls for “dynamic partnership” between oncologists and cardiologists^16^ and the creation of cardio-oncology practice guidelines promoting patient education of CVD risks^17,18^. Research has shown CVD risk factors such as obesity, hypertension, and inactivity are higher among cancer survivors than the general adult population^15,19^. Because many of these chronic risk factors require daily behavioral effort to manage, patient awareness and activation regarding management of these risks is especially important^20^. As the survivorship period lengthens and standard survivorship plans have ended^15^, patients may forget risk information, become generally less active^4^, and face other barriers to prevention behaviors^21^.

Cardio-oncology focused patient education, if it occurs, is most visible at discharge (e.g., after surgery)^22,23^, in part due to the need to avoid “information overload” during oncology treatment^24,25^. But there is limited research on survivor knowledge of CVD risks^15,26–28^ and essentially no understanding of patient retention of information over the survivorship continuum^29^. Optimal educational content, mode, and timing for cardio-oncology education across survivorship are also obscure^30,31^. Long-term (>5 year) survivors may require novel educational strategies because learning preferences change with age^32,33^, time since treatment^34^, technological skills^35^, and other psychological^36–38^ and behavioral variables^26,31,39^. Further research is needed to understand survivor self-assessed awareness, information needs^40^, and concerns^41,42^to promote beneficial lifestyle and behaviors^43,44^ across a potentially long survivorship continuum.

To understand cancer survivors’ self-assessed awareness of CVD risk and their information needs and preferences, we conducted a mixed method study with survey and interview components. The current report summarizes thematic findings from qualitative interviews conducted with predominantly long-term (>5 year) participants in community and virtual settings. Survey results are reported elsewhere. Our overall goal in this study was to characterize survivors’ current self-reported knowledge of CVD risks and best strategies for addressing their informational needs throughout the survivorship lifespan.

## Methods

### Design.

Using a phenomenological approach^4,45^ to survivor experience and perceptions of risk^46^, with interview questions secondarily attentive to socioecological theory’s^47,48^ multiple influential levels of patient contexts (from individual to societal), we conducted a qualitative study as part of a larger mixed methods study. We used semi-structured interviews in community or video conference settings. Objectives of the interviews were to assess survivor (a) awareness of their cardiovascular risks (whether specific treatment related risks, if any, or general concerns about obesity, hypertension, or inactivity), (b) engagement in prevention behaviors, and (c) preferences for health education.

### Setting.

The larger mixed methods study was an online survey with participants recruited through our community-based cancer survivor organizations (50 Hoops and the Oak Cliff Bible Fellowship), social media announcements, and direct emails to registered patients in the University of Texas Southwestern Medical Center cardio-oncology clinic. The sample for this qualitative interview study was drawn from participants recruited through more than 14 educational and screening events with our partner, community-based cancer survivor organizations in a historically underserved area of southern Dallas. These included tabling sessions at 2 large community health fairs, video lectures to community groups on cardio-oncology topics, and several presentations of our work in community settings. This mix of strategies^49^ enabled a diversity in the gender, age, race/ethnicity, and types of cancer of our participants while reflecting the demographic make-up of this sector in our service area.

### Participants.

Interview participants were drawn from the larger study sample and met the same inclusion criteria, namely: adult, English-speaking persons, recruited through the partner organizations, who had been diagnosed with any form of cancer.

We invited 26 participants enrolled in our larger, mixed-methods study to complete interviews. This sample size is not only adequate for identifying a high number of themes in qualitative analysis, but sufficient to capture all variance of meaning within themes.^50^ Participants were eligible for interview if they had completed the survey methods and lived in the Dallas area zip code. However, word-of-mouth recruitment through our southern Dallas partner produced a sample predominantly from southern Dallas.

Interview participants ranged in age from 30–82 (with an average age of 64.5 years), were majority African American (65%), and reported a prior diagnosis of the following cancers: breast (n=11); prostate (7); lung (2); stomach (1); melanoma (2); pancreatic (1); bladder (1); and colon and prostate (1). The majority of our sample (n=16) were also long-term survivors, having completed cancer treatment 10 or more years ago, while 6 had completed treatment within the last 5 years, and 4 were still in active treatment. (See Table 1)

### Data Collection.

Following an informed consent procedure that was approved by the UT Southwestern Medical Center Institutional Review Board (STU# 2023–0839), interviews lasted between 20 and 60 minutes, were recorded, then transcribed verbatim. Interviews were conducted by CSM, MP or another skilled research assistant (HH, see acknowledgements) under the supervision of CSM, a PhD medical anthropologist and licensed professional counselor. Interviews were held in quiet and private locations selected by the participants, including church spaces or in their homes via video conference. In consultation with our multi-disciplinary investigative team of qualitative research specialists, clinicians, and community members, CSM designed the Interview Guide informed by phenomenological^45,46^ and socioecological theoretical perspectives^47,48^ and based on knowledge of the cardio-oncology literature. Two pilot interviews were conducted to refine questions and sequencing. The final guide is provided in Table 2.

### Data Analysis.

Transcripts were analyzed in two phases by CSM, MP, or other skilled research assistants under the supervision of CSM (HH and KR, see acknowledgements). First, we employed framework analysis^51^ on a selection of interview responses to understand patterns across the sample on interval (e.g., How many years since cancer diagnosis?) and ordinal (e.g., Who talked to you about post-treatment care?) content in narrative responses. Second, we performed systematic coding of the transcribed narratives which produced a codebook of 12 codes: 7 parent and 5 child codes. (See Table 3) At least two independent coders used NVivo software^52^ to code the first 13 transcripts. At this point, inter-rater reliability scores reached .7^53^, the codebook was considered final (i.e., no new open coding or hierarchy changes), and a single coder was used thereafter. Additional post hoc coding was made to organize large code sets. Final analysis to determine major themes was made via inductive and deductive analysis of patterns and salience or importance to research goals^54,55^. That is, our systematic analysis of the coded data included triangulation^54,56^ of various data points (i.e., within code applications, content of the narratives themselves, and code co-occurrences) and the use of both inductive and deductive reasoning to recognize patterns in the data set.

## Themes

Our analysis produced three major themes: (1) relying on doctors to raise/address CVD risks; (2) CVD as a (non-)factor in oncology decision-making; and (3) learning preferences for CVD risk information. We report each in turn below.

### 1. Relying on Doctors to Raise/Address Cardio-Oncology Risks

We asked a series of questions about participants’ receipt and knowledge of information related to CVD risk, particularly risks related to their oncology treatments (See Table 1). We were concerned not with an objective test of knowledge, but with participants experience and self-assessed awareness of risks, whether specific to their own treatment, if any, or their general concerns about obesity, hypertension, or inactivity.

In general, participants thought their awareness of CVD risk information was high. In the earliest part of the interview, when we asked directly whether the understood their cancer treatment-related risks for CVD, most participants expressed they had been well informed by their clinicians, felt able to ask questions and make informed decisions about their cancer treatment risks and effects, and expressed satisfaction with their treatment. However, later questions probed for detail about participant knowledge of their CVD risk, and this narrative content revealed greater complexities including confusion and some revision to their original responses.

More specifically, in response to our question #3, “Did a health professional talk to you about your post-oncology treatment needs?”, 19 of 26 participants (73%) affirmed that their clinician had spoken with them. However, only 17 participants (65%) affirmed that they knew their risks for post-oncology CVD. fewer still (16 or 62%) said they understood the possible impacts of their oncology treatments to their heart. And many of their responses were very general about CVD risk (e.g., “I’m not aware of any.”).

These results likely reflect some social desirability response^57^. But the narratives convey participant confusion on this topic.

INTERVIEWER Do you think your cardiovascular risk changed after your cancer treatment?

INT04: No, not particularly, because I feel fine.

INT06: No, I’ve not had any cardiovascular risk. … Well, it was Dr. --- and I had another doctor who I don’t visit at all but she was my oncologist and she just said that it’s possible that I might get weakness of the heart or that kind of thing, but that they would monitor me and everything like that, that from the chemo and the radiation treatments that I got, that I might suffer some small thing. But they didn’t make a big deal of it.

Finally, when we asked, “What do you know about the impacts and risks of your cancer treatments?”, only 12 participants (46%) could clearly indicate their awareness of CVD risks with information or specific narratives about risk from their doctor. These participants were usually under the care of a doctor (often a cardiologist) and actively monitoring their CVD risk. Their responses conveyed a high level of trust that their doctor would know their risk and provide the right recommendations when the time comes, as in this example:

INT12: I don’t know where the risks are for my heart. If the risks were more in radiation, [I would have learned about them] right before we made that plan.

INT16: Well, if there had been any risks associated with my form of treatment, I think my surgeon would have mentioned them to me as well as my cardiologist, because I did have to get cardiology approval or prior to the surgery.

Clearly, knowledge of oncology-related CVD risk is a complex and evolving subject; one that even one nurse-patient did not fully understand:

INT04: I didn’t know. I, of course, know about breast cancer, but I didn’t know the different types of [it]. And, you know, because I was told that with my cancer, the triple negative, a lot of African American females get that, get this type. It doesn’t mean that I knew everything because I’m a nurse. I didn’t, you know? Or else, I probably did, and just, it just went out the window somewhere, you know?

Given this complexity, our phenomenological, narrative data convey a known challenge in post-oncology patient education. We asked participants to describe their receipt and knowledge of information related to CVD risk, particularly risks related to their oncology treatments. The semi-structured interview format, along with active probing by our interviewers, helped reveal differences between participants’ first responses (often “yes” to knowing their oncology-related CVD risks) and later, more elaborated narratives suggesting less complete knowledge. In the end, only 12 of 26 (46%) individuals clearly described their CVD risks in any detail and knew whether they were related to their oncology treatment. And patterns across narratives revealed high participant trust in their doctors, and a relative lack of oncology-related CVD risk knowledge among those who were not under active supervision of a doctor.

### 2. CVD as (Non-)Factor in Oncology Decision-Making

The second major theme addresses whether CVD risks factored into participants’ oncology decisions. We did not ask explicitly why participants underwent certain treatments. Instead, we captured participant views about the relative importance of CVD risks in their oncology treatment decisions. Most participants (n=20) said that CVD knowledge or lack of it would not have changed their decision to undergo cancer treatment. They described having had “no choice” due to the urgency of the situation or their primary concern being to survive the cancer, and that all other concerns being secondary or “irrelevant”.

INT02: But literally like this is like life or death and I wanted it out of my body. My doctors were like, we need a I need the data so I can make an informed decision and we need to act like immediately because again, we didn’t know if this lump had appeared like two months ago or was it six months ago.

INT04: They didn’t give me a chance to even think about an option.

INT08: I was like between a brick and a hard place. I didn’t want to cancel the spread. My only option -- I didn’t have another option.

INT10: All I was concerned [about was] I’ll get the cancer out.

INT15: I just wanted a treatment for the prostate cancer. As of that point in time, I didn’t put into consideration of my heart.

This urgency in many oncology cases may have been a substantial but reasonable barrier to better patient understanding of oncology-related CVD risks. But whether education was provided later, after oncology treatment, we take up in the next section.

Thus, only a few participants said that oncology-related CVD risks might have been a factor on their oncology treatment decisions. Their narratives suggest that greater attention to patient education may have made a difference to them, as to this man:

INT09: I’m not sure if it would have made a difference in my choice to take the prostate gland out. The only option we had was with radiation seed implants, watching/wait, or some of the freezing thing. Unless they said having prostatectomy would leave you with a damaged heart or something, then I would obviously considered that. But there was never a part of the discussion. …I’ve learned the hard way that side effects are certainly important to ask about.

One woman with breast cancer pointed out a lack of communication between her different doctors. She felt that the overlaps between the different specialties was not discussed with her:

INT14: I had the option of a lumpectomy, or mastectomy, which is either take it, just take the cancer out or take everything. …Everything else wasn’t really optional. … But sometimes I feel like they don’t really communicate with each other… Like my surgeon only talked about surgery. My oncologist doctor only talked about like the oncology part, the cancer part of it. My radiation doctor only talked about the effects of the radiation. Like, they never really crossed. When they spoke to me about things, they just stuck with their part of the treatment. …

But while this participant felt her doctors did not communicate with each other, she found the potential for overlapping concerns to be too complex for her to think about:

INT14: Don’t tell me anything because I don’t wanna have to change my mind. …Ohh it was enough, like I had enough to think about without having to consider something else, so not knowing for me was better because you know?

Only 2 participants said that additional information “maybe/possibly” would have led them to a different decision about oncology treatment. For example:

INT24: I think because if maybe they would have maybe gave me an in depth knowledge about the risk and that would have maybe influenced my decision.

And only one participant said “definitely”, they would have wanted to know but were not told by their oncologist.

INT20: Oh, I didn’t. No one mentioned anything about heart damage as a result of having that type of cancer or other procedures. No, I never heard of anything like that. … Well, if it’s gonna be some heart damage, definitely, I would have considered some other treatment.

Finally, 3 participants did not clearly answer this question. Overall, these data make clear that “too much information” is an important concern for many patients facing oncology treatment, especially if their cancer is deemed by physicians to require urgent action.

### 3. Learning Preferences for CVD Risk Information

We posed a number of questions to understand the learning preferences of our participants for information about oncology-related CVD risk. (See Table 4)

Fifteen (58%) reported that a conversation with their health professional was their preferred source of CVD risk information, and most were satisfied with the information they received.

INT24: It was an impressive conversation which I was able to express myself to the person as to how. And I was also getting a kind of emotional comfort and a word of advice from the person. … I prefer meeting the person in a one-on-one conversation, that will give the patient that comfort.

INT25: So it felt like a [inaudible] communication will be better so that they could actually detect my mood, know the atmosphere in which I was surrounded and know the best way to digest me and make me feel comfortable going for the surgery.

And when asked the ideal time to bring up information about oncology-related CVD risk, most (n=17) participants (65%) preferred these conversations to happen *before* oncology treatment began:

INT19: As far as the cancer, [the] doctor was not forthcoming in all the risks. So, I think more information, at least so we could be prepared. You know, even if he did go through the treatment the way he did, at least knowing more of what to expect.

INT03: So, for me it was because I could just hit it, and just go with it and at the same time just boom, boom, boom. Because I wanted to know everything I could about what was going on with me.

Six participants wanted conversation at multiple time points, to compensate for their own poor memory or concentration when they were fearful about the cancer diagnosis. For example:

INT13: And then when I finished, I would like to hear it again. So that would have given me a chance to have it at the initial stage, and I probably would not have heard it very well but I would have heard something. Again, when I went for therapy, [it] would have been reiterated and then it would have been probably on my frontal lobe, I would have remembered. And then before my dismissal, it would have been a good time to have it again. And then that way I could connect those pieces.

INT23: Somehow they should break some of this down, like even after you should have a post visit to talk about all these things.

Participants expressed preferences for how they would like to receive CVD information. A majority (n=23) preferred conversation or 1:1 with their provider. Ten also liked written materials, 6 requested online, video or telephone formats, and only 2 mentioned in-person, community-based events as a preferred format. The sub-group (n=10 of 26, or 39%) wanted written materials suggested these as a supplement to 1:1 conversations:

INT13: I would like it in a one to one personal [conversation]. And then giving it to me and in writing so I could read it later. I think a lot of times.

INT22: I would have preferred it in a different format like after the one-on-one conversation probably maybe get a hard copy or a soft copy PDF file of everything. Because it was a long conversation, so most of the things I might forget it.

And one suggestion in particular, for a personalized and hardbound book about their condition and their treatments, stood out among our participants’ narratives, given the lifelong need for information of many cancer survivors:

INT12: The other thing that I got, and now what I’m going to do is I’m going to go back and look to see if I just didn’t read that section… but I was blown away that I got in the mail like a bound book. They had my name on the front, my on-call or my surgeon’s name and picture on the front of the book…. It’s like 300 pages. It’s a bound proper book.

Finally, there was substantial variation in learning needs and preferences over time, depending on their anxiety about receipt of a diagnosis, the duration of treatment, and their life expectancy following treatment, as these participants explain:

INTERVIEWER: Did you prefer to know about your risk of cardiovascular when deciding, like when they were telling you about treatments.

INT04: I really wasn’t interested in that at that time.

INT09: No, I’ve had no discussion about that [oncology-related CVD risk]. The only time I’ve heard about that is when [de-identified] and when you were doing this study. And so we’ve been working with them with different things. So, that’s the first I’ve heard that there might be a connection between cancer and cardiovascular.

INT05: Your mind is not fully open to what they are telling you. Sometimes, initially you’re thinking, you are going to die. And so now, once you get past that stage, well, maybe you …can process the information that you’re receiving.

## Discussion

Given the apparent inconsistencies in CVD risk knowledge uncovered over the course of our interview - that is, the fact that 19 participants (73%) initially declared they knew their CVD risks but ultimately only 12 (46%) could clearly describe their own CVD risks in any detail -

A systematic analysis of narratives from 26 majority long-term (>10 year) cancer survivors to understand their CVD risk knowledge and learning preferences yielded three thematic findings. First, differences between participants’ initial self-assessments of risk awareness and later narratives which lacked clarity about such risks were not surprising and may be partially attributable to social desirability influence^57^. However participants’ expressions of high reliance on doctors to bring risks to their attention when necessary^58–61^ suggests to us a low level of activation^20,62^ or a de-activated stage of change.^63^ Providers may have inadequate time, educational facilities, or knowledge to facilitate adequate patient education^64,65^. Thus, survivor reliance on physicians alone, without their own behavioral and lifestyle prevention efforts, may contribute to survivors’ greater risk for CVD. An opportunity exists for cardio-oncology prevention education and/or activation among survivors^66,67^.

Second, participants were satisfied with the information they received surrounding their oncology treatment and conveyed that one could have “too much information” when facing an oncology treatment decision. Thus, CVD risk education may be better place in the post-treatment period to avoid “information overload”^24,25^. Factors such as timing of education during or after oncology treatments^68,69^, readiness for learning^70,71^, and learning preferences for format and content^30,31^ also affect survivor learning. Further, because of the increasing duration of cancer survivorship, diverse educational strategies may be required at different times in the survivorship period^41,72^ in order to account for some of the unique barriers faced by long-term survivors^21,38^.

Third, while conversation with one’s healthcare team was the favored learning format of most participants, several would have preferred conversation at multiple points in time including before and after treatment. Such preferences can place a high burden on providers{Yi, 2025, Primary care providers and their needs caring for cancer survivors: a qualitative study;Birken, 2019, Survivorship care plan implementation in US cancer programs: a national survey of cancer care providers}. Almost half of our majority-long-term survivors also wanted written materials for later consultation. Durable written materials, such as personalized bound books detailing one’s own diagnosis and treatment, may be especially useful for long-term survivor health literacy and patient-provider communications^73,74^. Our results also indicate survivor interest in online and virtual materials, which may alleviate some of the burden on providers to address this education and activation need.

Finally, results of this study corroborate the limited reports on patient experience of shock and anxiety over learning of the risk or detection of CVD^66^. Our qualitative data also provide narratives expressing patient concern over lack of information on cardiotoxicity, their need for health education, and a lack of collaboration between oncologists and cardiologists^67^.

Overall, survivors’ high sense of risk awareness coupled with a relatively poor ability to discuss risks (whether specific treatment related risks, if any, or general concerns about obesity, hypertension, or inactivity) suggests an opportunity to improve survivor awareness and, possibly, preventive behaviors. Improving survivor CVD knowledge may support better communication with healthcare teams and survivor participation in cardio-oncology prevention and treatment^29,75^.

### Limitations

Our participants were predominantly survivors of breast or prostate cancers, and a predominantly African American sampled from a limited geographic area. These factors limit the generalizability of our findings to other cancer types or other groups. Also, while our phenomenological study design produced diverse perspectives on survivor risk awareness and learning preferences, we did not objectively test participants’ CVD risk knowledge.

## Conclusion

This mixed methods study of cancer survivor’ knowledge of CVD risk and their educational needs and preferences yielded important and actionable details about survivor health literacy, and their diverse learning needs and preferences over potentially long survivorship periods. To inform more effective patient education, further research is needed to design and test effective health literacy strategies for long-term survivors at high risk for CVD.

## Figures and Tables

**Table 1.** Sample Characteristics.

Participant Characteristic	Results
Total	26
Age	Range 30–82
	Avg. 64.5
Gender	Male 11
	Female 15
Ethnicity	Black (17)
	White (2)
	Unreported (7)
Cancer types (self-reported)	Breast (11)
	Prostate (7)
	Lung (2)
	Stomach (1)
	Melanoma (2)
	Pancreatic (1)
	Bladder (1)
	Colon and prostate (1)
Time since completion of cancer treatment (self-reported)	Still active (4)
	5 years (6)
	10 years or more (16)

**Table 2.** Interview Guide

Interview Guide
Tell me about your understanding of your risks for cardiovascular disease? What do you know about the impacts and risks of your cancer treatments? Did your cardiovascular risk change after your cancer treatment? Yes/No Who, if anyone, explained this to you? If so, how did it change? When you were under treatment for your cancer, did any health professionals talk to you about your post-treatment needs? Was the impact of your cancer treatment on your heart discussed? By whom? What do you remember learning? How was the information conveyed to you? Would you have preferred it in a different manner/format, time or from a different person? What is your biggest concern today about the changes you may need to make for your health? Can you please discuss how you felt learning new information about your health risks when you were going through cancer treatment. How do you feel about learning new information about your health risks now? Enjoy, avoid, etc. What should clinicians know about your process of learning? Your preferences for when, where, by whom? As a cancer survivor, do you approach your health differently now? Please explain.

**Table 3.** Codebook with Definitions and # of Unique Excerpts.

Code	Definition	# Unique Excerpts
Changed Risk (for CVD)	Discussions about their changed CVD risk, or participant reaction to learning about their potentially higher CVD risk; may include learning early or late about these dangers; may include no change in CVD or CVD was not discussed as the opposite of changed CVD.	207
Knowledge	Participant narratives about general disease knowledge (e.g., other risk factors, family history), shared knowledge (e.g., experience of others going through treatment). Existing awareness and understanding of their CVD risk, lack of risk knowledge, absence of knowledge.	121
Format	Passages that address the medium (e.g., pamphlets, conversation with provider, other), language, delivered by (e.g., which healthcare provider).	105
Agency	Discussions about actively (with agency) participating in any treatment decision-making, including asking questions (of any health care personnel), or seeking information from any source; a demonstration of agency or participation in decision-making.	101
Reaction (to Diagnosis, Treatment)	Reaction to or experience of oncology treatment in general; can include emotion or practical actions; this code can remain very broad.	83
Content	Passages that indicate information desired (e.g., more info about race and cancer); also used for lack of content (absence of info) from a given source or opportunity.	70
Timing	Passages about when they received (or prefer to receive) information; could be a time of day, or during an appointment; anything that marks the timing of receiving information.	68
Factor (in decision-making)	Passages in which a participant describes something, including CVD knowledge, as a factor in their decision to undergo oncology treatment, or decision about which oncology treatment to undergo.	67
Stage of Treatment	Narratives about the current or past status of their cancer treatment, or how long ago it was; recent or in the remote past; any time-related indications of readiness for information.	59
Confusion	Narrative about confusion due to absence of knowledge about disease, risk, treatment options, etc.; feelings due to the absence of knowledge.	46
Surprise	Participants surprised associated with their oncology treatments or CVD risk; focus is on patients being under-informed within the context of getting cancer; should not refer to emotion/feelings generally about receiving a cancer diagnosis.	21
Rush	Any passages that indicate rushed decisions; inability to ask all the questions one wanted to ask.	18

**Table 4.** Learning Preferences.

**How was the information about CVD risk conveyed to you?**	
Conversation w health professional	15
Online research	1
Pamphlets from clinic	2
It was not conveyed to me	8
**Would you have preferred it in a different manner or format?**	
Conversation/more conversation	7
More written material/details	5
Webinar	1
No/Was satisfied with format	13
**When is it best to bring up this information about oncology-related CVD risk?**	
Before treatment	17
Multiple times/Before, during, and/or after treatment	6
Not asked/No answer	3
